# Transient inhibition of microsomal prostaglandin E synthase-1 after status epilepticus blunts brain inflammation and is neuroprotective

**DOI:** 10.1186/s13041-023-01008-y

**Published:** 2023-01-25

**Authors:** Nelufar Yasmen, Madison N. Sluter, Lexiao Li, Ying Yu, Jianxiong Jiang

**Affiliations:** Department of Pharmaceutical Sciences, College of Pharmacy, The University of Tennessee Health Science Center, Memphis, TN 38163 USA

**Keywords:** Antiseizure drugs (ASDs), Epilepsy, Epileptogenesis, Reactive gliosis, Inhibitor, Microsomal prostaglandin E synthase-1 (mPGES-1), Neuroinflammation, Neuroprotection, Prostaglandin E2 (PGE_2_), Seizures

## Abstract

Status epilepticus (SE) in humans is characterized by prolonged convulsive seizures that are generalized and often difficult to control. The current antiseizure drugs (ASDs) aim to stop seizures quickly enough to prevent the SE-induced brain inflammation, injury, and long-term sequelae. However, sole reliance on acute therapies is imprudent because prompt treatment may not always be possible under certain circumstances. The pathophysiological mechanisms underlying the devastating consequences of SE are presumably associated with neuroinflammatory reactions, where prostaglandin E2 (PGE_2_) plays a pivotal role. As the terminal synthase for pathogenic PGE_2_, the microsomal prostaglandin E synthase-1 (mPGES-1) is rapidly and robustly induced by prolonged seizures. Congenital deletion of mPGES-1 in mice is neuroprotective and blunts gliosis following chemoconvulsant seizures, suggesting the feasibility of mPGES-1 as a potential antiepileptic target. Herein, we investigated the effects of a dual species mPGES-1 inhibitor in a mouse pilocarpine model of SE. Treatment with the mPGES-1 inhibitor in mice after SE that was terminated by diazepam, a fast-acting benzodiazepine, time-dependently abolished the SE-induced PGE_2_ within the brain. Its negligible effects on cyclooxygenases, the enzymes responsible for the initial step of PGE_2_ biosynthesis, validated its specificity to mPGES-1. Post-SE inhibition of mPGES-1 also blunted proinflammatory cytokines and reactive gliosis in the hippocampus and broadly prevented neuronal damage in a number of brain areas. Thus, pharmacological inhibition of mPGES-1 by small-molecule inhibitors might provide an adjunctive strategy that can be implemented hours after SE, together with first-line ASDs, to reduce SE-provoked brain inflammation and injury.

## Introduction

Status epilepticus (SE) is the second most common neurological emergency and is symptomized by one prolonged unintermittent seizure or several seizures without full return to baseline [1]. Depending on studies and resources, SE in general affects 10–40 people per 100,000 population each year, among which generalized convulsive SE constitutes approximately 45–74% of all cases, and the overall rate of mortality associated with SE is about 20–30%. Moreover, nearly 23% patients who survive SE will deteriorate in neurological functions at some points [2]. The current antiseizure drugs (ASDs) aim to stop seizures quickly enough to prevent high mortality and morbidity. However, short response time is critical for the efficacy of ASDs and can be challenging in some clinical settings. Currently, the median delays to the first-line treatment are around 30–70 min [3]. Thus, adjunctive treatment that can be given well after the SE initiation to diminish the long-lasting outcomes of SE is in urgent demand.

The pathophysiological mechanisms underlying the devastating consequences of SE remain largely elusive. However, several lines of evidence support that SE-promoted powerful inflammatory reactions within the brain engage cytokines and many other proinflammatory mediators to aggravate neuronal death and exacerbate neuronal hyperexcitability, leading to unprovoked seizures, behavioral deficits, and pharmacoresistance [4–8]. Among epilepsy-associated proinflammatory mediators that have been identified during the past two decades, prostaglandin E2 (PGE_2_) can be rapidly and robustly induced by prolonged seizures and, in turn, essentially contributes to neuropathogenesis in the epileptic brain via acting on four G protein-coupled receptors EP1-EP4 [9, 10]. To synthesize PGE_2_, membrane-released arachidonic acid is first converted to an intermediate called prostaglandin H2 (PGH_2_) by cyclooxygenase (COX), which has two isoforms: COX-1 and COX-2. The short-lived PGH_2_ is then catalyzed to PGE_2_ by three prostaglandin E synthases, i.e., microsomal prostaglandin E synthase-1 (mPGES-1), mPGES-2, and cytosolic PGES (cPGES). Among these, COX-2 and mPGES-1 are inducible isozymes and contemporaneously upregulated by acute neuronal insults such as seizures and strokes [9, 11]. Functionally coupled to COX-2, mPGES-1 directly converts COX-2-derived PGH_2_ to PGE_2_ [12], suggesting that mPGES-1 might represent an alternative target to COX-2 for anti-inflammatory therapeutics.

Genetic ablation of mPGES-1 decreases neuronal damage following intrahippocampal injection of kainic acid (KA) in mice and reduces glutamate release from hippocampal slices treated by KA [13, 14], indicating a role of mPGES-1 in seizure-promoted excitotoxicity. The deficiency of mPGES-1 also leads to decreased vulnerability of mice to pentylenetetrazol-induced seizures and blunts the seizure-provoked gliosis in the hippocampus [15]. In addition, inhibiting mPGES-1 in humanized mPGES-1 mice by a human mPGES-1 inhibitor BI1029539 reduces KA SE-induced upregulation of P-glycoprotein and activity at the blood–brain barrier [10]. However, to date, pharmacological inhibition of mPGES-1 has not been systemically evaluated in wildtype animals after SE. This is likely because most current mPGES-1 inhibitors are developed for the human ortholog and do not block the rodent mPGES-1 due to the interspecies difference in the enzyme structures [16]. Utilizing a recently reported selective small-molecule inhibitor of mPGES-1, *N*-phenyl-*N'*-(4-benzyloxyphenoxycarbonyl)-4-chlorophenylsulfonyl hydrazide (PBCH), which is potent and can effectively inhibit both human and rodent orthologs [17, 18], we herein aim to determine the feasibility of pharmacologically targeting mPGES-1 in wildtype mice following pilocarpine-induced SE. Our results demonstrate the feasibility of mPGES-1 as a promising pharmacological target for delayed adjunctive treatment that could reduce neuropathogenesis triggered by SE.

## Materials and methods

### Chemicals and compounds

Methylscopolamine (Cat. # S2250), terbutaline (Cat. #T2528), and pilocarpine (Cat. #P6503) were purchased from Sigma-Aldrich. Parenteral diazepam (Hospira, Lot #30561) was purchased from Henry Schein. Indomethacin was purchased from Tocris Bioscience (Cat. #1708) and celecoxib was purchased from Cayman Chemical (Cat. # 10008672). Fluoro-Jade B (Cat. #AG310) was purchased from Sigma-Aldrich. Compound PBCH was kindly provided by Dr. Jae Yeol Lee [17, 18] and authenticated using LC/MS and NMR in the Medicinal Chemistry Core at the University of Tennessee Health Science Center.

### Experimental animals

Young adult male C57BL/6 mice (~ 8 weeks old) were purchased from Charles River Laboratories, housed under a 12-h light/ dark cycle in standard relative humidity at room temperature, and provided free access to food and water. All animal procedures were approved by the Institutional Animal Care and Use Committee (IACUC) of the University of Tennessee Health Science Center and carried out carefully in full compliance with the Guide for the Care and Use of Laboratory Animals (Eighth Edition) provided by the National Institutes of Health (NIH).

### Pilocarpine model of SE and drug treatment

Mice were pre-treated with methylscopolamine and terbutaline (2 mg/kg each in saline, i.p.) to minimize the unwanted effects of pilocarpine in peripheral organs. Thirty minutes later, pilocarpine (280 mg/kg in saline, freshly prepared, i.p.) was administered to induce seizures in mice. Seizures were classified using a modified Racine scale as we previously described [19, 20]. 0: normal behavior—grooming, walking, exploring, and sniffing; 1: immobile, staring, jumpy, and curled-up posture; 2: automatism—repetitive blinking, head bobbing, chewing, vibrissae twitching, face-washing, scratching, and “star-gazing”; 3: partial body clonus, sporadic myoclonic jerks, and shivering; 4: full body clonus, “corkscrew” turning and flipping, loss of posture, rearing, and falling; 5: onset of SE—nonintermittent seizure activities; 6: bouncing, wild running, and tonic seizures; 7: death. SE was defined by nonintermittent seizure activities and usually indicated by continual generalized clonic seizures without returning back to low-stage seizures. SE proceeded for 1 h and was terminated by diazepam (10 mg/kg, i.p.). Two hours after SE onset, that is, 1 h after administration of diazepam, animals were randomized for treatment with either compound PBCH (10 mg/kg, i.p.) or vehicle (10% DMSO, 50% PEG 400, 40% ddH_2_O). Mice were treated again at 8 and 20 h after SE onset. During recovery from SE, mice were fed moistened rodent chow, monitored daily, and given 5% dextrose in lactated Ringer’s solution (Baxter) when needed. Four days after SE, animals were euthanized under deep anesthesia with isoflurane and perfused with cold PBS to wash blood out of the brain. The hippocampal and cortical tissues were then collected for biochemical and histological analyses.

### PGE_2_ measurement

PGE_2_ in the brain tissues was measured using the PGE_2_ Multi-Format ELISA Kit (Arbor Assays, Cat. # K051). A total of 50 µL diluted lysate of each cortical sample was used for PGE_2_ measurement following the manufacturer’s protocol as we previously described [9, 21]. The absorbance was measured using a Synergy H1 microplate reader (BioTek) at 450 nm. A standard curve for PGE_2_ was run with each experiment. The PGE_2_ levels were normalized to tissue weights for comparisons between experimental groups.

### COX inhibition assay

The COX Colorimetric Inhibitor Screening Assay Kit (Cayman Chemical, Cat. #701050) was used to evaluate the inhibition percentage of COX enzyme by tested compounds following the manufacturer’s manual. Non-selective COX inhibitor indomethacin and selective COX-2 inhibitor celecoxib were used as control compounds. A plate reader (Synergy H1) was used to measure the absorbance at 590 nm.

### Quantitative PCR

The mRNA expression levels of examined genes were quantified by quantitative PCR (qPCR) as we described previously [9, 22]. The total RNA from mouse brain tissues was isolated using TRIzol and the PureLink RNA Mini Kit (Invitrogen). RNA purity and concentration were measured using A260/A280 ratio and A260 value measured by a microvolume spectrophotometer (NanoDrop One, Thermo Fisher). The complementary DNA (cDNA) was synthesized using the SuperScript III First-Strand Synthesis SuperMix (Invitrogen). The qPCR was performed using cDNA, primers, and 2 × SYBR Green SuperMix with a final reaction volume of 20 µL in a CFX96 Touch Real-Time PCR Detection System (Bio-Rad Laboratories). Cycling conditions were set as: 95 °C for 2 min followed by 40 cycles of 95 °C for 15 s and then 60 °C for 1 min. Melting curve analysis was utilized to validate the specificity of the PCR products. Fluorescent data were obtained at the 60 °C step. The cycle of quantification for GAPDH was subtracted from the cycle of quantification measured for each gene of interest to yield ∆Cq. Samples that did not undergo cDNA synthesis served as negative controls. The sequences of primers used for qPCR are as follows: *CCL2*, forward 5’-CATCCACGTGTTGGCTCA-3’ and reverse 5’-GCTGCTGGTGATCCTCTTGTA-3’; *CCL3*, forward 5’-TGCCCTTGCTGTTCTTCTCT-3’ and reverse 5’-GTGGAATCTTCCGGCTGTAG-3’; *CCL4*, forward 5’-CATGAAGCTCTGCGTGTCTG-3’ and reverse 5’-GGAGGGTCAGAGCCCATT’; *GAPDH*, forward 5’-TGTCCGTCGTGGATCTGAC-3’ and reverse 5’-CCTGCTTCACCACCTTCTTG-3’; *GFAP*, forward 5’-GACAACTTTGCACAGGACCTC-3’ and reverse 5’-ATACGCAGCCAGGTTGTTCT-3’; *Iba1*, forward 5’-GGATTTGCAGGGAGGAAAAG-3’ and reverse 5’-TGGGATCATCGAGGAATTG-3’; *IL-1β*, forward 5’-TGAGCACCTTCTTTTCCTTCA-3’ and reverse 5’-TTGTCTAATGGGAACGTCACAC-3’; *IL-6*, forward 5’-TCTAATTCATATCTTCAACCAAGAGG-3’ and reverse 5’-TGGTCCTTAGCCACTCCTTC-3’; *TNF-α*, forward 5’-TCTTCTGTCTACTGAACTTCGG-3’ and reverse 5’-AAGATGATCTGAGTGTGAGGG-3’.

### Immunohistochemistry

Coronal brain sections (25 µm) were fixed by 4% paraformaldehyde and underwent 10-min permeabilization by 0.2% Triton X-100, followed by blocking with 10% goat serum in PBS for 60 min. The sections were then incubated in rabbit anti-Iba1 polyclonal antibody (Wako Chemicals, Cat. # 019-19741, 1:200) or rabbit anti-GFAP polyclonal antibody (Thermo Fisher, Cat. # PA1-10019, 1:500) at 4 °C overnight. Sections were then washed and incubated with anti-rabbit secondary antibody conjugated with Alexa Fluor 546 (Invitrogen, Cat. # A-11035, 1:1000) for 1 h at room temperature. After washing, sections were stained with DAPI for 10 min and carefully mounted onto slides using ProLong Gold antifade mountant (Invitrogen, Cat. # P36930). Digital images were captured using a BZ-X800 fluorescence microscope (Keyence), and the image processing and quantitative analyses were performed using the ImageJ/Fiji software (NIH).

### Fluoro-Jade B staining

Fluoro-Jade B (FJB) reagent is a neuron-specific anion which can selectively label degenerating neurons [23], and was used to detect SE-induced neuronal death in this study as we previously described [24–26]. In brief, coronal brain sections were successively immersed in 80% alcohol containing 1% NaOH for 5 min, in 70% alcohol for 2 min, and in distilled water for 2 min. Sections were then incubated in 0.06% potassium permanganate for 30 min with gentle agitation. After rinsing in distilled water for 1 min, sections were transferred to the FJB solution (0.0004, w/v, in distilled water with 0.1% acetic acid) for 30 min with gentle agitation in the dark. Sections were rinsed with three 1-min changes of distilled water, rapidly dried, and covered by the coverslip with DPX mountant. Images were captured by a BZ-X800 fluorescence microscope (Keyence), and the FJB-positive cells were counted in the sections between bregma − 1.5 and − 3.

### Statistical analysis

Statistical analyses were performed using GraphPad Prism software by *t* test or Mann–Whitney *U* test as indicated in each experiment. *P* < 0.05 was considered statistically significant. All data are presented as mean + or ± SEM.

## Results

**Fig. 1:**
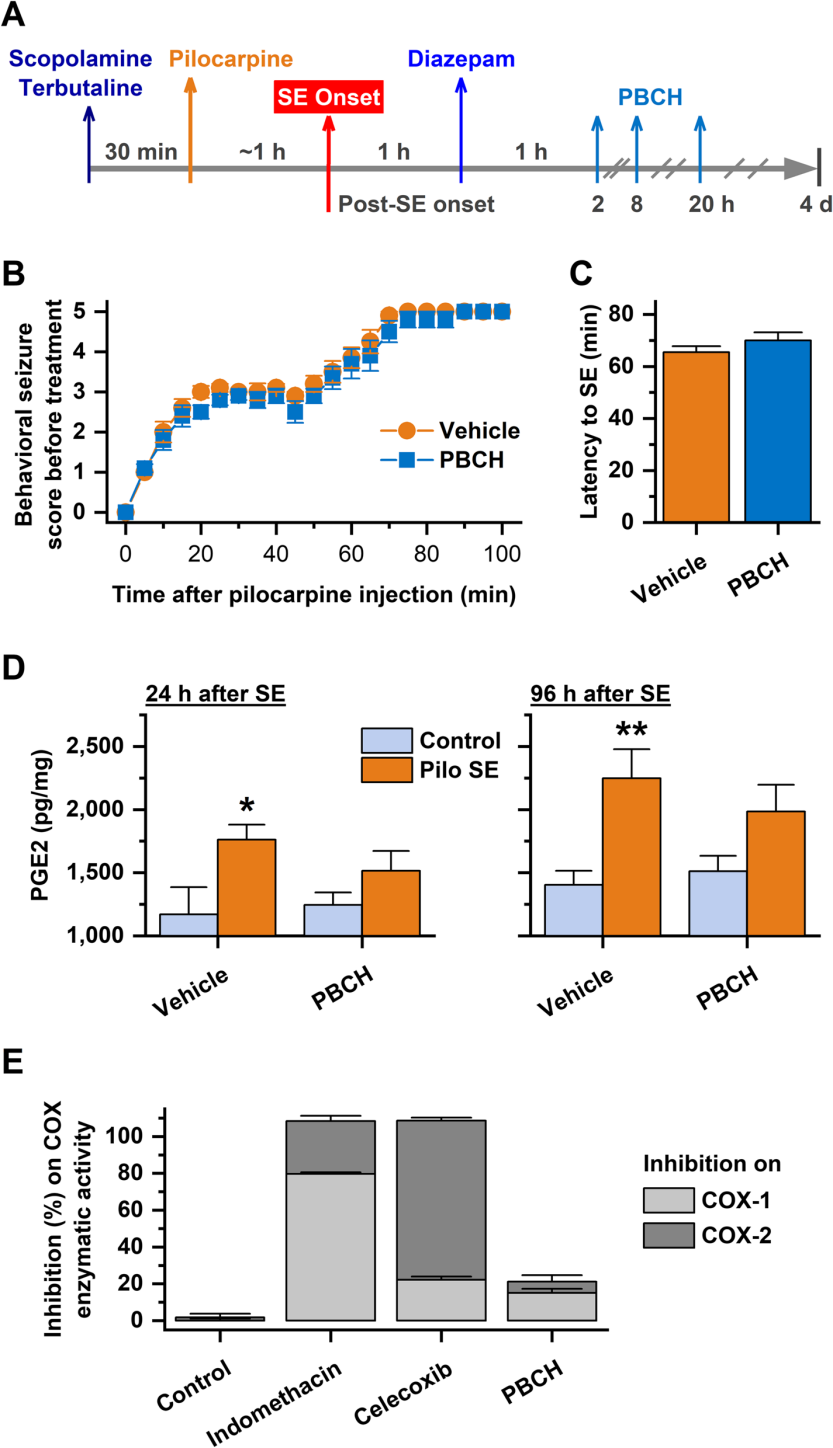
Pharmacological inhibition of mPGES-1 diminishes SE-promoted PGE_2_ in the brain. **A** Schematic diagram showing the timeline for experimental procedure and treatment. Mice were treated with methylscopolamine and terbutaline (2 mg/kg each, i.p.) and 30 min later by pilocarpine (280 mg/kg, i.p.) for SE induction. SE proceeded for ~ 60 min and was then interrupted by diazepam (10 mg/kg, i.p.). After recovery for 1 h, mice were randomly treated with either vehicle or compound PBCH (10 mg/kg, i.p.). Mice were treated again at 8 h and 20 h after SE onset. All animals were sacrificed 4 d after SE for neuropathological analyses. **B** Behavioral seizure scores were tabulated every 5 min prior to treatment with vehicle or PBCH (*N* = 10). **C** The latency to reach behavioral SE after pilocarpine administration (*P* = 0.2545, unpaired *t* test). **D** PGE_2_ levels in the hippocampus 1 d after SE (Left) and in the cortex 4 d after SE (Right) were measured by ELISA and normalized to tissue weights (pg/mg) for comparisons by Mann–Whitney *U* test. Left: *P = 0.0182 for vehicle groups; *P* = 0.3845 for PBCH groups. Right: **P = 0.0091 for vehicle groups; *P* = 0.6048 for PBCH groups. **E** COX inhibition assay was performed to assess the inhibition of compound PBCH on COX-1 and COX-2 with indomethacin and celecoxib as reference compounds (*N* = 3). All compounds were tested at 10 µM, a relatively high concentration aiming to achieve their full inhibitory potential on COX enzymes, which was demonstrated by indomethacin as a non-selective COX inhibitor and celecoxib as a selective COX-2 inhibitor. Data are presented as mean + or ± SEM

Acute seizures can quickly induce mPGES-1, which together with concurrently elevated COX-2, leads to a rapid increase in PGE_2_ levels in the brain [9, 27, 28]. Genetic ablation of mPGES-1 in mice is associated with decreased neuronal damage and neuroinflammation following chemoconvulsant seizures [13, 14], indicative of a pathogenic role of mPGES-1 in the epileptic brain. However, whether mPGES-1 is a feasible target for interrupting neuropathogenesis triggered by prolonged seizures remains questionable due to the lack of target validation using pharmacological agents. To answer this question, we assessed the effects of mPGES-1 inhibition by compound PBCH in a mouse pilocarpine SE model (Fig. 1A). Mice were first treated with scopolamine and terbutaline (2 mg/kg each, i.p.) to minimize the undesired effects of pilocarpine in the periphery [29]. Half an hour later, seizures were induced by systemic administration of pilocarpine (280 mg/kg, i.p.), and SE in mice typically began in about 60 min after pilocarpine injection. The SE was allowed to proceed for 1 h and then terminated by treatment with diazepam (10 mg/kg, i.p.). After recovery for another hour, mice that survived SE were randomized and treated with either vehicle or compound PBHC (10 mg/kg, i.p.), followed by two more doses that were given at 8 h and 20 h after the SE onset (Fig. 1A). The first two doses were timed to overlap the early peak of mPGES-1 induction in this model, whereas the third dose was designated to suppress mPGES-1 activity during the subsiding phase of its elevation [27].

There was no noticeable difference in either the time-course progression of convulsive seizures (Fig. 1B) or the latency for animals to reach behavioral SE (Fig. 1C) between vehicle and PBCH groups after pilocarpine administration, confirming that the animals from these two experimental groups had been effectively randomized prior to further treatment. It appears that an episode of 1-h SE was able to substantially increase the PGE_2_ levels by 51% in the hippocampus 1 d after SE (*P* < 0.05, Fig. 1D left). Even after 3 additional days, the PGE_2_ levels in the cortex remained elevated by 60% (*P* < 0.01, Fig. 1D right), suggesting a long-lasting increase in the mPGES-1 activity in different brain areas triggered by pilocarpine-induced seizures. However, the SE-elevated PGE_2_ in these brain areas was largely blunted by treatment with compound PBCH for only 3 times—at 2, 8, and 20 h after SE onset. This inhibitory effect by PBCH lasted for up to 4 d after SE albeit there was a trend that PGE_2_ regained increase once treatment stopped (Fig. 1D). Compound PBCH, when given at a relatively high concentration (10 µM) contrasting its nanomolar potencies [17, 18, 30], only had negligible effects on COX enzymatic activities in an assay where non-selective COX inhibitor indomethacin and selective COX-2 inhibitor celecoxib showed substantial inhibition as positive controls (Fig. 1E). These findings, taken together, suggest that the decrease in brain PGE_2_ by treatment with PBCH was likely attributed to its inhibition on mPGES-1 but not COX-1 or COX-2.

**Fig. 2:**
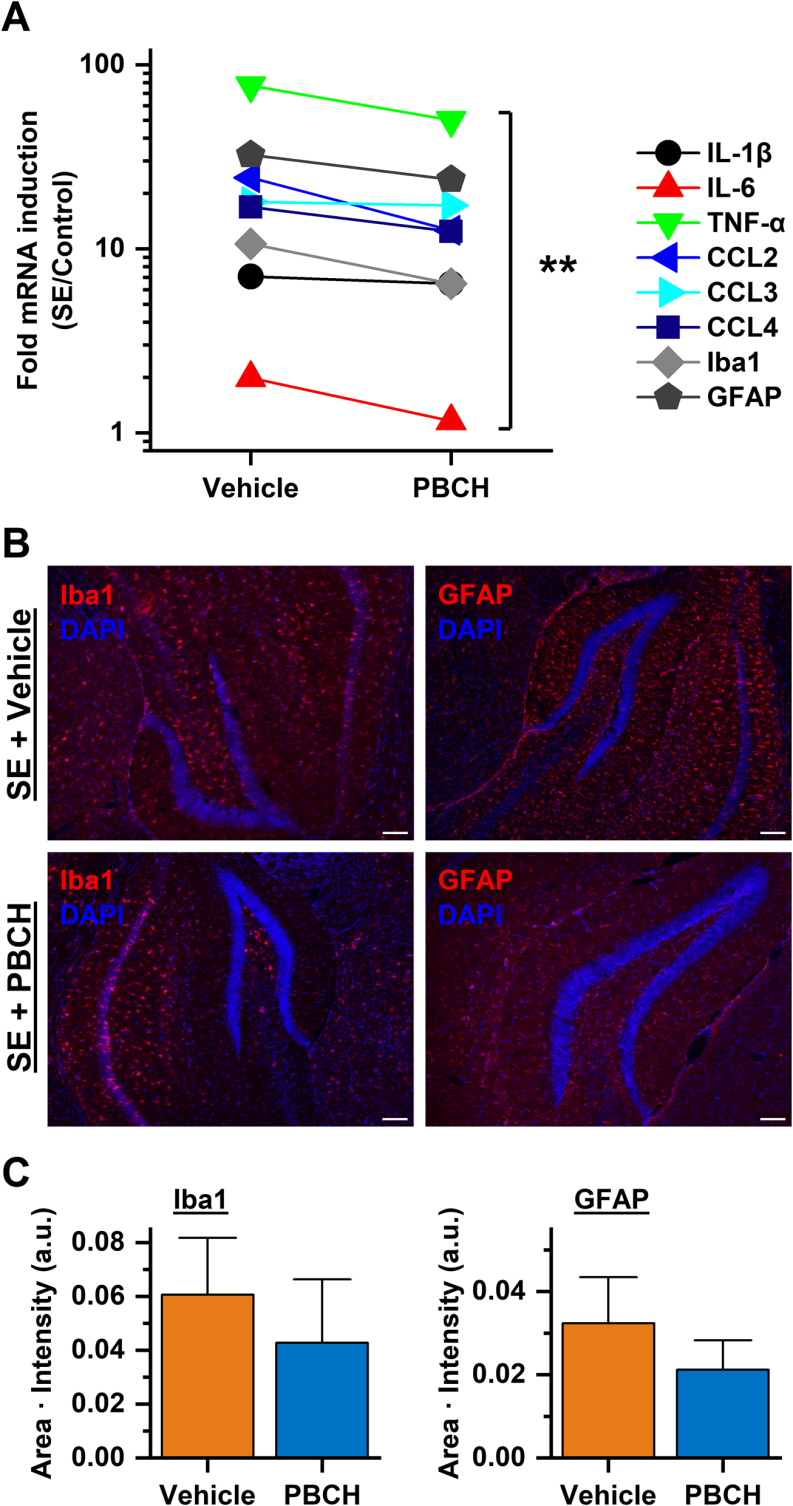
Blockade of mPGES-1 blunts post-SE neuroinflammation. **A** The mRNA expression of cytokines (IL-1β, IL-6, and TNF-α), chemokines (CCL2, CCL3, and CCL4), and gliosis biomarkers (Iba1 and GFAP) in the hippocampus was measured by qPCR 4 d after pilocarpine-induced SE in mice (*N* = 6, **P = 0.0021, ratio *t* test). **B** Immunostaining for Iba1 and GFAP was performed 4 d after SE to visualize reactive microglia and astrocytes in the hippocampus, respectively. The overall density of fluorescence (red) was used to indicate the levels of gliosis. Note that nuclear counterstain was performed with DAPI (blue fluorescence). Scale bar = 100 μm. **C** Quantification of the protein expression of Iba1 and GFAP in the hippocampi of SE mice that were treated by vehicle or PBCH (*N* = 6, *P* = 0.5842 for Iba1; *P* = 0.4135 for GFAP, unpaired *t* test). Data are presented as mean + SEM

Prolonged seizures can initiate several lines of neuroinflammatory processes that often precede the occurrence of spontaneous seizures and presumably play an essential role in epileptogenesis [31–34]. We next evaluated the effects of mPGES-1 inhibition by PBCH on SE-induced brain inflammation via examining the mRNA expression of a number of inflammation-associated genes in the hippocampus 4 d after pilocarpine-induced SE. It was found that the post-SE treatment with PBCH overall decreased the SE-promoted hippocampal expression of proinflammatory cytokines and chemokines including IL-1β, IL-6, TNF-α, CCL2, CCL3, and CCL4 (*P* < 0.01, Fig. 2A). Pilocarpine SE-induced neuroinflammation in the hippocampus was also featured by the proliferation and hypertrophy of glial cells including microglia and astrocytes, shown by the elevation of microglial biomarker Iba1 and astrocytic biomarker GFAP at both mRNA levels (Fig. 2A) and protein levels (Fig. 2B). However, the post-SE treatment with mPGES-1 inhibitor PBCH for only 3 doses decreased the SE-induced mRNA expression of Iba1 and GFAP in the hippocampus by 39% and 26%, respectively, measured by qPCR 4 d after SE (Fig. 2A). Consistently, with this transient treatment of PBCH, the protein levels of these two reactive gliosis biomarkers in the hippocampal areas measured by immunostaining at the same time were also reduced by 30% and 35%, respectively (Fig. 2C). These results together demonstrate that the seizure-induced mPGES-1 is involved in brain cytokine storm and reactive gliosis following SE via generating PGE_2_.

**Fig. 3:**
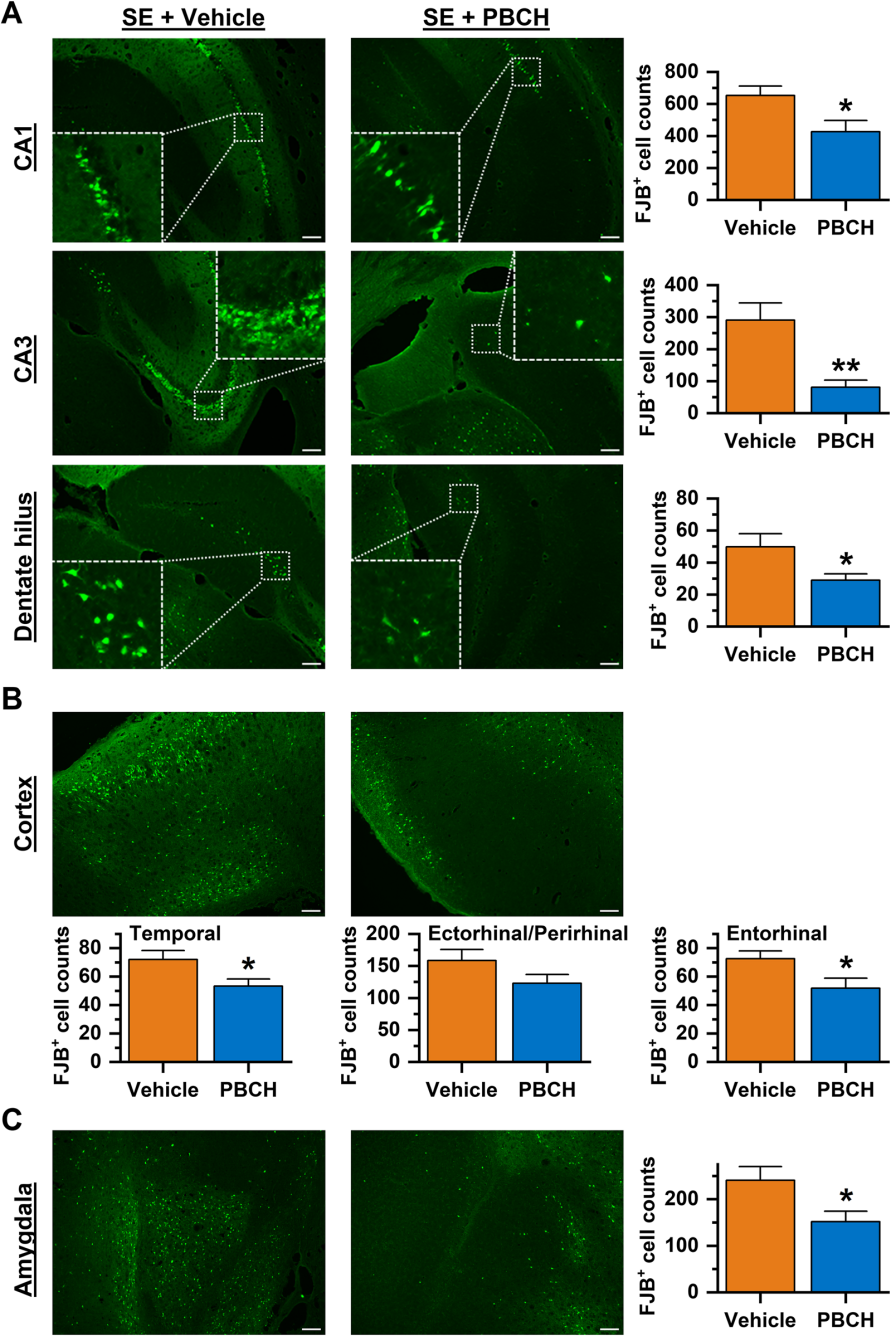
Post-SE mPGES-1 inhibition is neuroprotective. Neuronal injuries in the hippocampus (**A**), cortex (**B**), and amygdala (**C**) were visualized by Fluoro-Jade B (FJB) staining 4 d after pilocarpine-induced SE in mice that were treated by vehicle or compound PBCH. Scale bar = 100 μm. FJB-positive cells per section were counted in these brain areas and used for quantification of neurodegenerating neurons in each mouse brain for comparisons (*N* = 8, *P = 0.0275 for hippocampal CA1; **P = 0.0027 for CA3; *P = 0.0399 for dentate hilus; *P = 0.0367 for temporal cortex; *P* = 0.1314 for ectorhinal and perirhinal cortex; *P = 0.0349 for entorhinal cortex; *P = 0.0319 for amygdala, unpaired *t* test). Data are shown as mean + SEM

Sustained high-intensity seizures can kill neurons and may also trigger the subsequent unprovoked seizures; therefore, brain cell death is considered as an essential step of acquired epileptogenesis, particularly in the mature brain [35, 36]. We then next evaluated neuronal damage 4 d after pilocarpine SE. Coronal brain sections were prepared and stained with Fluoro-Jade B (FJB) reagent to label degenerating neurons, and the positively stained cells in each section were counted to indicate the injury level. Pilocarpine-induced SE for 1 h led to substantial neuronal damage in the vehicle-treated mice, illustrated by extensive FJB-positive cells throughout the hippocampus (Fig. 3A). However, the post-SE treatment with mPGES-1 inhibitor PBCH decreased the FJB-positive cells by 35% (*P* < 0.05) in the hippocampal CA1 area, 72% (*P* < 0.01) in the CA3 area, and 42% (*P* < 0.05) in the dentate hilus (Fig. 3A). In line with findings in hippocampus, treatment with PBCH prevented SE-triggered neuronal death by 26% (*P* < 0.05) in the temporal cortex, 22% in the perirhinal and ectorhinal cortex, and 29% (*P* < 0.05) in the entorhinal cortex (Fig. 3B). Likewise, mPGES-1 inhibition after pilocarpine SE also reduced FJB-positive cells by 37% (*P* < 0.05) in the amygdala (Fig. 3C). This widespread and significant neuroprotection observed in the broad brain areas demonstrates that the mPGES-1 inhibition is a highly effective strategy to protect neurons from SE-induced brain damage.

## Discussion

A number of previous studies demonstrated that the congenital deletion of mPGES-1 in mice provided extensive benefits in several chemoconvulsant models, such as neuroprotection, reduced susceptibility, and blunted neuroinflammation [13–15]. In the present study, we comprehensively evaluated the therapeutic effects of blocking mPGES-1 by a dual species inhibitor PBCH in a mouse pilocarpine model of SE. To the best of our knowledge, this is the *first* pharmacological study on mPGES-1 in wildtype animals that experienced prolonged seizures. We found that systemic administration of PBCH after pilocarpine SE for three times was sufficient to abolish the SE-promoted PGE_2_ in the brain. This was likely caused by a direct inhibition of PBCH on mPGES-1 because the compound did not significantly block either COX-1 or COX-2. Moreover, mPGES-1 inhibition by PBCH also blunted several key proinflammatory cytokines and reactive gliosis in the hippocampus and largely prevented neuronal damage in a broad range of brain areas.

As the inducible COX isozyme, COX-2 is responsible for the first step in the biosynthesis of PGE_2_ following prolonged seizures, and thus was widely considered as a promising target for anti-inflammatory therapies treating seizures and epilepsy [6, 37, 38]. However, in addition to PGE_2_, the elevation of COX-2 enzymatic activity also leads to the production of the other four prostanoids, namely, prostaglandin D2 (PGD_2_), prostaglandin F2α (PGF_2α_), prostaglandin I2 (PGI_2_ or prostacyclin), and thromboxane A2 (TXA_2_), which together can mediate a myriad of beneficial and detrimental effects in both the CNS and periphery depending on their responding receptors and cellular context [39–41]. Indeed, COX-2 inhibition by selective inhibitors (Coxibs) or non-steroid anti-inflammatory drugs (NSAIDs) yielded controversial results in several animal seizure models [6, 37, 38]. The first two decades of this century also witnessed the growing concerns over the adverse effects of Coxibs and NSAIDs on microvessels that are associated with disturbing risks in life-threatening vascular incidents [42]. As such, targeting a specific PGE_2_ synthase, i.e., the inducible mPGES-1, presumably does not affect other types of prostanoids, and thus might provide an alternative anti-inflammatory strategy for the delayed treatment of seizures with more specificity than imprudently shutting down the entire COX cascade by taking Coxibs or NSAIDs.

PGE_2_ exerts diverse physiological and pathogenic functions via signaling through four membrane-bound receptors (EP1-EP4) that have divergent engagement in G protein-dependent and independent signaling pathways [43–45]. These four PGE_2_ receptor subtypes have been studied intensively for their potential as new targets for seizures in several chemoconvulsant models. Particularly, EP1 receptor activation by seizure-induced PGE_2_ might aggravate seizure severity via increasing brain excitability and contribute to drug-resistance in epilepsy due to its positive modulation on P-glycoprotein expression [10, 39]. On the other hand, PGE_2_ signaling via EP2 promotes neuroinflammation and neuronal injury in the hippocampus and causes subsequent behavioral deficits in several chemoconvulsant models [24, 25, 28, 46, 47]. Likewise, EP3 and EP4 receptors have also been demonstrated to contribute to some aspects of chemoconvulsant seizures [39]. As such, modulating a single PGE_2_ receptor subtype likely will leave some therapeutic room for others. In contrast, selectively targeting mPGES-1 by small-molecule inhibitors like PBCH would prevent detrimental effects mediated by all four PGE_2_ receptor subtypes without affecting other COX-derived biolipids, such as prostacyclin and thromboxane that are essential to maintaining the hemostatic balance [48]. Its low basal expression, high inducibility, and unique position in the COX cascade together support mPGES-1 as an ideal druggable target for a more delicate balance between therapeutic efficacy and specificity than the upstream COX enzymes and the downstream PGE_2_ receptors [40, 41, 49, 50].

In sum, the present work, for the *first* time, suggests that the pharmacological inhibition of the inducible terminal enzyme for PGE_2_ biosynthesis after generalized convulsive SE diminishes neuroinflammation and prevents neuronal death in wildtype mice. Our findings together support that mPGES-1 represents a feasible and likely more specific target, as an alternative to COX-2 and PGE_2_ receptors, for delayed adjunctive treatment, along with current first-line ASDs such as diazepam, lorazepam, and midazolam, for the management of prolonged seizures and the associated pathogenic alterations in the epileptic brain.

## Data Availability

Data and materials will be made available based on reasonable request.

## Abbreviations

ASDs: Antiseizure drugs
a.u.: Arbitrary unit
CCL2: Chemokine (C-C motif) ligand 2
CCL3: Chemokine (C-C motif) ligand 3
CCL4: Chemokine (C-C motif) ligand 4
COX: Cyclooxygenase
Coxibs: Selective COX-2 inhibitors
cPGES: Cytosolic prostaglandin E synthase
DAPI: 4’,6-Diamidino-2-phenylindole
ELISA: Enzyme-linked immunosorbent assay
FJB: Fluoro-Jade B
GFAP: Glial fibrillary acidic protein
Iba1: Ionized calcium binding adaptor molecule 1
IL-1β: Interleukin 1β
IL-6: Interleukin 6
i.p.: Intraperitoneal injection
KA: Kainic acid
mPGES-1: Microsomal prostaglandin E synthase-1
mPGES-2: Microsomal prostaglandin E synthase-2
NSAIDs: Non-steroid anti-inflammatory drugs
PBCH: *N*-Phenyl-*N'*-(4-benzyloxyphenoxycarbonyl)-4-chlorophenylsulfonyl hydrazide
PGD_2_: Prostaglandin D2
PGE_2_: Prostaglandin E2
PGF_2α_: Prostaglandin F2α
PGH_2_: Prostaglandin H2
PGI_2_: Prostaglandin I2 or prostacyclin
qPCR: Quantitative PCR
SE: Status epilepticus
TNF-α: Tumor necrosis factor α
TXA2: Thromboxane A2
